# A comparative life cycle assessment of stretchable and rigid electronics: a case study of cardiac monitoring devices

**DOI:** 10.1007/s13762-021-03388-x

**Published:** 2021-05-26

**Authors:** S. Kokare, F. M. A. Asif, G. Mårtensson, S. Shoaib-ul-Hasan, A. Rashid, M. Roci, N. Salehi

**Affiliations:** 1 Department of Production Engineering, KTH Royal Institute of Technology, Brinellvägen 68, 100 44 Stockholm, Sweden; 2 Department of Protein Science, KTH Royal Institute of Technology, Mycronic AB Nytorpsvägen 9, 183 03 Täby, Sweden

**Keywords:** Life cycle assessment, Stretchable electronics, Printed circuit board, Cardiac monitoring device, Electrical and electronic equipment

## Abstract

Stretchable electronics is a new innovation and becoming popular in various fields, especially in the healthcare sector. Since stretchable electronics use less printed circuit boards (PCBs), it is expected that the environmental performance of a stretchable electronics-based device is better than a rigid electronics-based device that provides the same functionalities. Yet, such a study is rarely available. Thus, the main purpose of this research is to perform a comparative life cycle analysis of stretchable and rigid electronics-based devices. This research combines both the case study approach and the research review approach. For the case study, a cardiac monitoring device with both stretchable and rigid electronics is used. The ISO 14044:2006 standard's prescribed LCA approach and ReCiPe 2016 Midpoint (Hierarchist) are followed for the impact assessment using the SimaPro 9.1 software. The LCA results show that the stretchable cardiac monitoring device has better environmental performance in all eighteen impact categories. This research also shows that the manufacturing process of stretchable electronics has lower environmental impacts than those for rigid electronics. The main reasons for the improved environmental performance of stretchable electronics are lower consumption of raw material as well as decreased energy consumption during manufacturing. Based on the LCA results of a cardiac monitoring device, the study concludes that stretchable electronics and their manufacturing process have better environmental performance in comparison with the rigid electronics and their manufacturing process.

## Introduction

The production and consumption of electrical and electronic equipment (EEE) in the European Union (EU) are on the rise (Eurostat 2020). Due to low levels of reuse, collection, recycling, and other forms of recovery of waste EEE, the consumption of rare and expensive natural resources is also increasing. This imposes higher economic and environmental pressure on manufacturers of modern electronic devices. Most of these devices include printed circuit boards (PCBs) as an essential component. The manufacturing of PCBs is a complex and energy-intensive process (Bogdanski et al. 2013). Furthermore, the materials used in PCBs are hazardous, can be expensive, and are extremely difficult to recycle or recover value in a profitable and sustainable way (Long et al. 2016). The separation process of the electronic components often makes the components unusable because of the temperature applied (Canal Marques et al. 2013). Currently, a majority of the PCBs contained in electronic wastes are incinerated, which causes environmental damage, as well as health and safety issues for humans (Kumar et al. 2018). To achieve better environmental performance, electronics manufacturers are searching for and implementing new manufacturing methods and technologies to reduce both the material used to produce the PCBs and the energy demand of the manufacturing process (Esfandyari et al. 2015). One such emerging technology is stretchable electronics (He et al. 2015; Khan et al. 2020).

As new fields of applications for stretchable electronics continue to emerge, such as wearable smart textiles and medical/health-monitoring devices, the market for stretchable electronics is expected to grow rapidly. Amid the COVID-19 crisis, research indicates that the global stretchable electronics market will reach $2.6 billion by 2027 (Researchandmarkets 2020). Assuming that the market for stretchable electronics grows as predicted, it becomes an interesting topic for research to understand the environmental impact of stretchable electronics and their manufacturing process compared to the traditional rigid ones. This is particularly interesting for original equipment manufacturers (OEM) that produce advanced production machines, which are used for the manufacturing of electronics, and want to understand the environmental impacts of the manufacturing processes and their tools. Thus, the main objective of this research is to assess and compare the environmental impacts of stretchable electronics and rigid electronics with a particular focus on their manufacturing processes.

The paper is structured in the following sections. Section “Method” describes the LCA method, while “Literature review on LCA of stretchable electronics” section provides a review of existing LCA studies on stretchable electronics. Section “Case study” describes the case study including the design and different life cycle phases of stretchable and rigid cardiac monitoring devices. Section “Life cycle assessment of cardiac monitoring devices” presents different phases of the LCA study of cardiac monitoring devices. Section “Results, discussion and sensitivity analysis” presents the results of the comparative life cycle analysis and their interpretation, while “Conclusion” section presents the main conclusions of the study together with some ideas about future research. This research was conducted between January 2020 and June 2020 in Stockholm, Sweden.

## Method

Life Cycle Assessment (LCA) is a useful method to identify and quantify the environmental impacts of a product, process, or activity. Comparative LCA can be carried to compare the environmental impacts of two or more products that have similar functionality. To perform the proposed comparative LCA, this research combines both the case study approach and the research review approach. Here, a case study of a cardiac monitoring device is used to compare the environmental impacts of devices based on stretchable and rigid electronics with a special focus on the manufacturing processes for the PCBs. A cardiac monitoring device was chosen since it is readily available and is representative of a large number of physiological monitoring devices. The conclusions drawn from the analysis of the manufacturing process should be analogous to a class of related products using stretchable electronics. For the impact assessment, the LCA approach prescribed by the ISO 14044:2006 standard is used (ISO 2006). According to ISO 14044: 2006 standard, LCA is carried out in the following steps:

1. Goal and Scope Definition: Identifying and determining the aim of the study, system boundaries, functional unit, impact assessment method, impact categories, assumptions, limitations, etc.
2. Life Cycle Inventory (LCI) Analysis: Collecting the inventory data like raw materials, energy consumed, environmental releases, etc., associated with each life cycle stage. The sources for this inventory data include different LCA databases, scientific literature, reports from public authorities, etc.
3. Life Cycle Impact Assessment (LCIA): Modeling of inventory data collected in LCA software package and translating the environmental emissions into environmental impact categories using the characterization factors of the LCA method selected.
4. Interpretation: Analyzing the results of impact assessment; identifying different environmental hot spots; and making recommendations to minimize the environmental impacts.

ReCiPe 2016 Midpoint (Hierarchist) is used as the impact assessment method. The commercial LCA software package used for this study was SimaPro 9.1 (PRé Sustainability 2019).

## Literature review on LCA of stretchable electronics

For the research review, the five-step review process suggested by Creswell (2002) is followed. The review process is used for the purpose of exploration to gain insights about the relevant work performed previously (Leedy and Ormrod 2015), and setting the scope of the case study, as well as outlining relevant assumptions where data are not available.

The research review shows that a very limited number of studies have previously looked into the environmental impacts of stretchable electronics or their comparison with rigid electronics. In fact, most of the LCA studies assess the environmental impacts of consumer electronic devices, like personal computers and laptops, mobile phones, televisions, electronic media, white goods, and other domestic appliances (Subramanian and Yung 2016). Apart from those studies found concerning consumer electronic devices, a small number of studies have been identified that looked into the environmental footprints of photovoltaic cells, integration technologies in microelectronics and electric vehicles (Andersen et al. 2014).

Concerning the stretchable electronics, Kunnari et al. (2009) conducted a preliminary LCA study of a printed wristband fabricated using an inkjet printing process and conventional lithography. The results showed that the optimization of the inkjet printing process could result in consuming less electricity than a traditional lithographic process. Similarly, LCA studies performed by Kanth et al. (2011, 2012) concluded that printed polymer substrate-based RFID technology is more sustainable than conventional rigid PCB technology. Espinosa et al. (2012) conducted an LCA of indium tin oxide (ITO)-free solar cells and compared it with ITO-based solar cells. Furthermore, Liu et al. (2014) carried out a comparative LCA of conventional organic-based printed circuit boards (O-PCBs) and paper-based printed circuit boards (P-PCBs) and recommended the use of P-PCBs for reducing the environmental impacts. Wan (2017) demonstrated through LCA that inkjet-printed flexible cables for ECG monitoring systems are more eco-friendly than the rigid cables. Ahmed et al. (2017) found that roll-to-roll manufactured modules of stretchable triboelectric nanogenerators (TENGs) are more environmentally friendly than the traditional photovoltaics. This study considered raw materials and energy consumed in their fabrication while neglecting the disposal phase. Ma et al. (2017) studied if a wearable smartwatch is a “green product” using LCA and energy-dispersive X-ray (EDX) spectroscopy.

From the review, it is evident that the environmental impact assessment of stretchable electronic devices is not widely covered in the literature. Furthermore, the literature did not consider the influence of the manufacturing process of stretchable electronics on the environmental impacts. This review confirms that comparing the life cycle impacts of the stretchable and rigid electronics and their associated manufacturing processes is a novel study.

## Case study

This research is part of the EU-funded research project SINTEC (Soft Intelligence Epidermal Communication Platform).^1^ SINTEC has received funding from the European Union’s Horizon 2020 research and innovation program under Grant Agreement No 824984. The project aims to develop soft, stretchable smart sensor patch technology, as well as a suitable, commercially viable manufacturing process, and demonstrate its advantages over conventional rigid electronics technology for this class of devices. One of the main application areas for stretchable electronics technology is in the realm of physiological monitoring for personal and professional reasons. The development of this technology involves the use of novel materials and manufacturing processes. Thus, from an environmental perspective, the sustainability of stretchable electronics devices, as well as the processes used to manufacture them, needs to be assessed with respect to the manufacturing of the conventional rigid electronics devices. Since physiological monitoring is identified as one of the central application areas for stretchable electronics technology, a cardiac monitoring device is chosen for the comparative analysis due to its relatively simple design, as well as its availability.

### Cardiac monitoring devices and their manufacturing processes

#### Life cycle of a rigid cardiac monitoring device

**Fig. 1:**
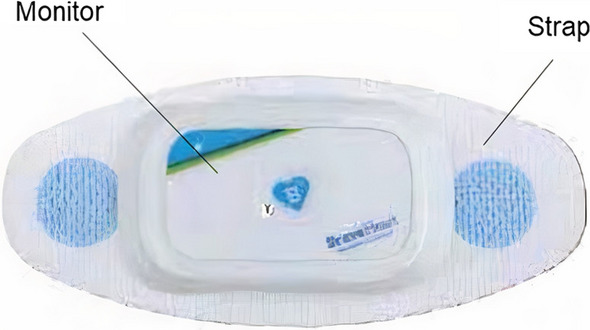
A rigid cardiac monitoring device (courtesy: BraveHeart Wireless, Inc.)

The rigid cardiac monitoring device consists of a monitor and a strap as shown in Fig. 1. The strap is a self-adhesive patch that is worn on the skin. It is made of polyurethane and an acrylic adhesive. The interconnections of silver nitrate are printed on this strap. The monitor is mounted on the strap. The monitor consists of the outer casing made of ABS (acrylonitrile butadiene styrene) polymer that contains a battery and electronic components, included in the PCB, inside it.

The raw material for the rigid cardiac monitoring device is extracted from nature. The manufacturing phase involves the production of individual components of the monitor and the strap, as well as their assembly. The manufacturing of the cardiac monitor includes injection molding of ABS to form the outer casing, manufacturing of the PCB from printed wiring boards (PWB) and semiconductor devices using surface-mount technology (SMT), and drawing of metallic wires for sensor electrode. The SMT process for the manufacturing of the PCB consists of the deposition of solder paste on the PWB, an inspection of the solder paste deposition, mounting of components, an inspection of component mounting, and finally, a reflow step, see Fig. 3a. In the reflow process, the PCB is carried through an oven with a material-specific temperature profile that melts solder paste that has been deposited on the substrate to create a conductive and structural connection between a component and the substrate. The mounted PCB is inserted into a molded case to produce a complete monitoring unit. The strap is manufactured using a roll-to-roll printing process. The monitor and strap are assembled and shipped to the user. The product is used by the user, and after its useful life, it is discarded by the user. In the end of life (EoL) phase, some parts of the product are recycled, while others are incinerated.

#### Life cycle of a stretchable cardiac monitoring device

**Fig. 2:**
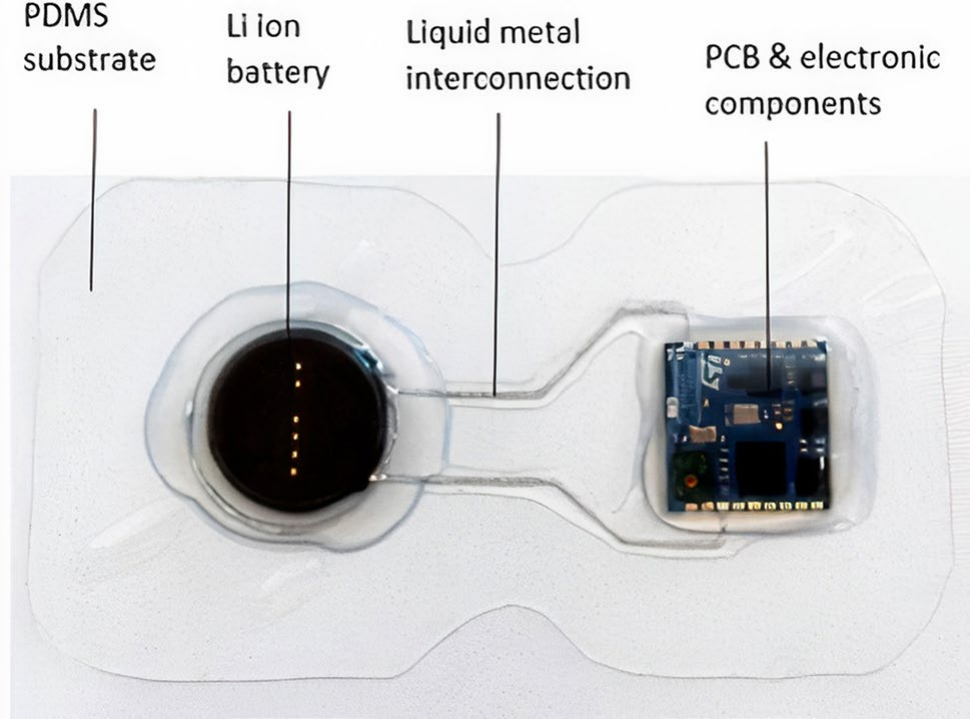
A development model of a stretchable cardiac monitoring device (SINTEC project)

The stretchable cardiac monitoring device (shown in Fig. 2) used in this study consists of a lithium-ion battery, liquid metal interconnections, and a sensor component embedded on a soft stretchable substrate made of PDMS (polydimethylsiloxane) polymer. The liquid metal alloy (LMA) used here is Galinstan, a eutectic alloy of gallium, indium, and tin (Indium Corporation, New York, USA^2^). The stretchable cardiac monitoring device is lightweight and integrates better with human skin in comparison with the rigid device. It should be noted that the stretchable cardiac monitoring device described in the article is developed as a prototype device in the SINTEC project and is not commercially available. The PCB used in the stretchable device is smaller than the rigid device and has fewer embedded electronic components, integrates circuits on the polymeric substrate, and connects individual parts on the device using LMA.

**Fig. 3:**
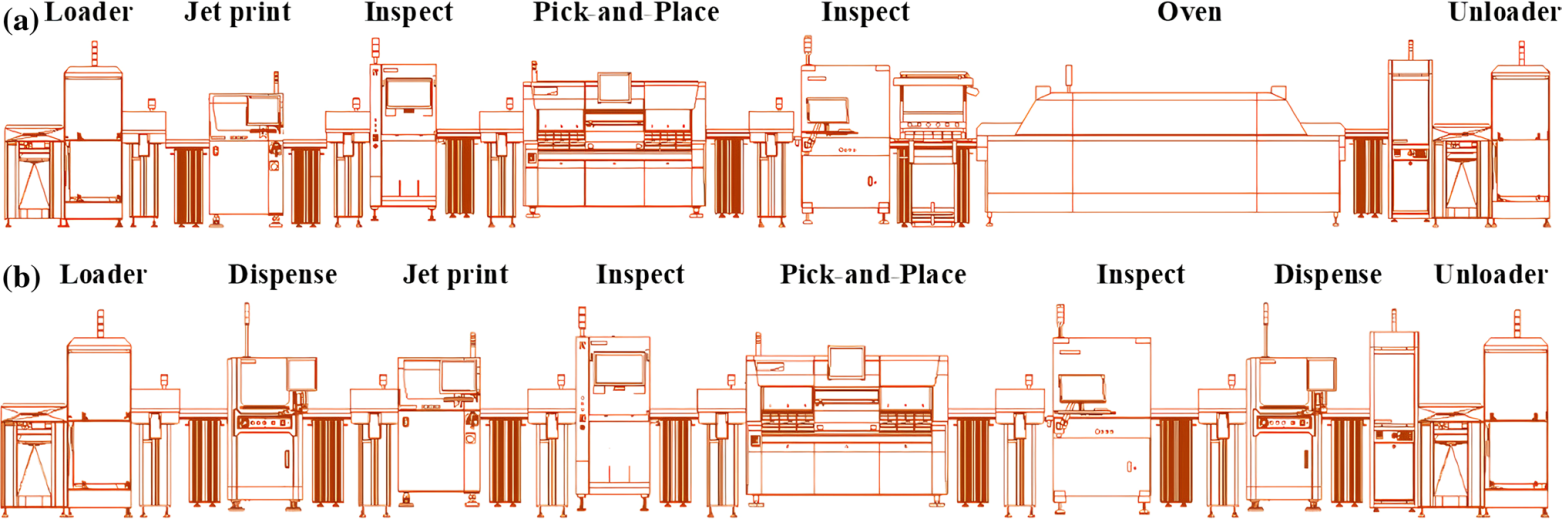
Schematic of a) manufacturing of PCB for a rigid device that is then used in the subsequent manufacturing of the cardiac monitor and b) manufacturing of stretchable device with integrated PCB

The life cycle of the stretchable cardiac monitoring device begins with the extraction and synthesis of raw materials from nature. The manufacturing of this device involves establishing a stretchable base substrate, followed by the deposition of liquid metal on the substrate, placement of advanced components, battery and semiconductor devices on the substrate, and the deposition of a stretchable coverage layer. The process also includes inspection stages to control material deposition and component mounting. A schematic of the manufacturing process is shown in Fig. 3b. The product is used by the user and disposed in the EoL phase. In the end of life (EoL) phase, some parts of the product are recycled, while others are incinerated.

Table 1 summarizes the steps associated with the manufacturing of rigid and stretchable cardiac monitoring devices. Due to the design of the products, it is evident that the manufacturing steps and associated processes are different. These process differences influence the environmental impacts of the devices along their complete life cycle and in particular during the manufacturing phase. This is further discussed in the next section.

**Table 1:** Summary of the manufacturing steps and associated processes for rigid and stretchable cardiac monitoring devices

Rigid cardiac monitoring device		Stretchable cardiac monitoring device	
*Manufacturing step*	*Description*	*Manufacturing step*	*Description*
Manufacturing of casings	Injection molding of ABS	Loader	Loading of raw materials
Manufacturing of wires	Wire drawing of copper	Dispenser	Deposition of PDMS substrate
Strap fabrication	Roll-to-roll printing of strap	Jet printing	Deposition of Galinstan
Loader	Loading of raw materials	Inspection	Visual inspection
Jet printing	Deposition of solder paste	Pick & place	Deposition of electronic components
Inspection	Visual inspection	Inspection	Visual inspection
Pick & place	Deposition of electronic components	Dispense	Deposition of PE encapsulation layer
Inspection	Visual inspection	Unloader	Unloading of the final product
Oven	Reflow		
Unloader	Unloading of the final product		

## Life cycle assessment of cardiac monitoring devices

### Goal and scope definition

The goal of this study is to assess and compare the environmental impacts of the stretchable cardiac monitoring device with those of the rigid device. The purpose of the comparative study is to understand which device and its associated manufacturing process is more sustainable. This understanding may support decisions concerning the design, manufacturing, and use of a conventional rigid cardiac monitoring device or a device based on stretchable technology in order to take advantage of environmental performance along with other user-related differences.

### Functional unit and system boundaries

**Fig. 4:**
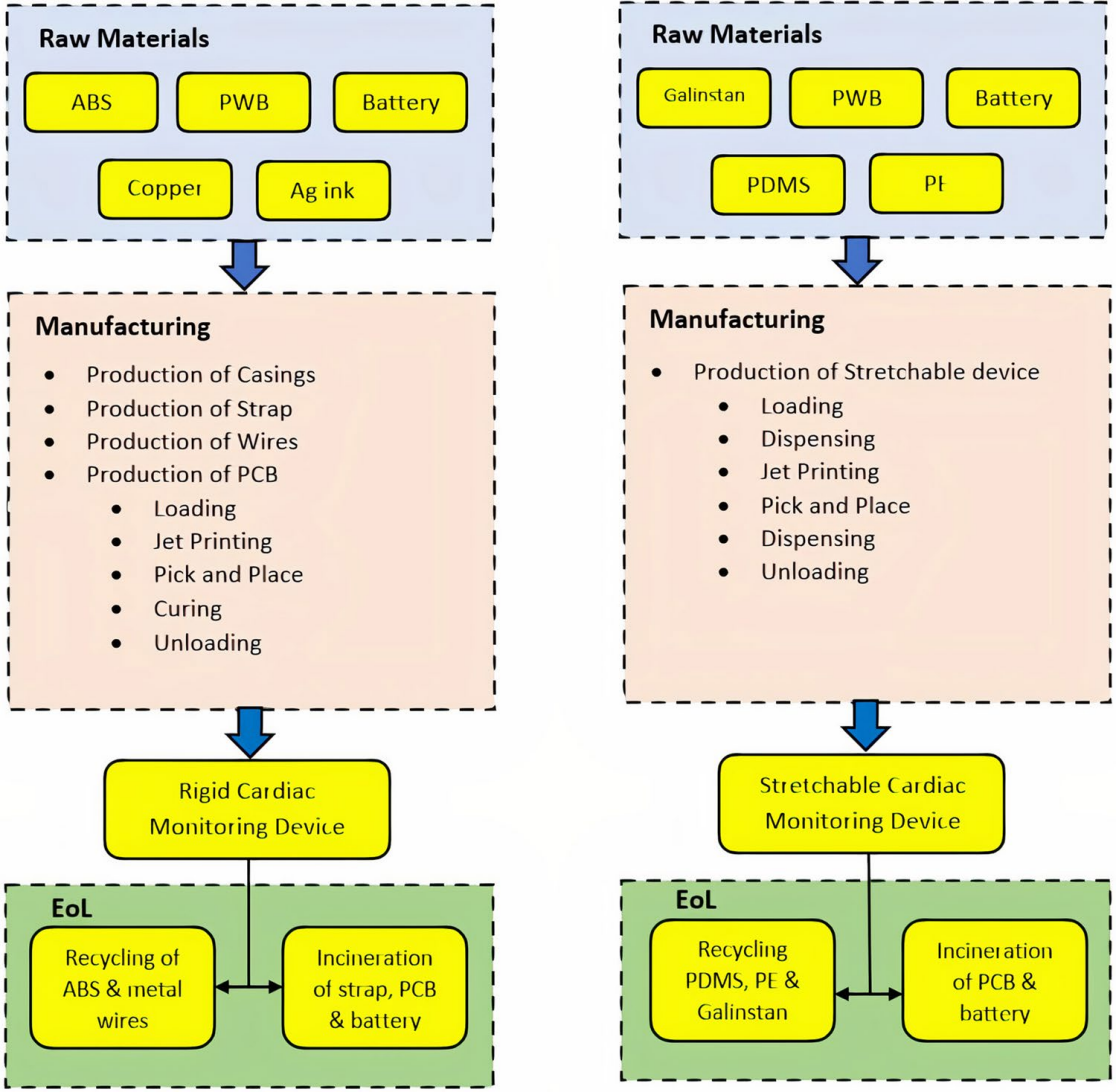
System boundaries of rigid (left) and stretchable (right) cardiac monitoring devices

The functional unit provides a reference to which the input and output inventory flows are related (Curran 2015). Both cardiac monitoring devices have the same functionality. The functional unit considered in this study is one unit rigid and one unit stretchable cardiac monitoring device. The system boundary and the scope of this study are shown in Fig. 4 and include phases for raw material extraction, manufacturing, and end of life. This study does not include the use phase due to the fact that the use phase in this case primarily involves the energy consumed from the batteries used in both devices, which assumed to be identical. Both devices use a non-rechargeable lithium-ion battery power rating of 3 V, 80 mAh. Since the batteries are identical, it is assumed that the environmental impacts during the use phase of both devices are the same and, therefore, do not contribute to this comparative study.

The transportation of the raw materials from the suppliers and the products to the customers has also been excluded from this study. This is because the stretchable device is not yet commercially available and, therefore, the raw material suppliers, as well as the distribution channels, are not finalized.

A distinction between foreground and background processes is made. The data related to foreground processes, i.e., the manufacturing processes specific to this study, collected from Mycronic AB, whereas the data connected to background processes, i.e., generic processes, are taken from the LCA software’s database for raw materials, some manufacturing processes (production of casings, strap, and wires), and end of life phases.

### Life cycle inventory analysis

The complete lifecycle inventory data for the rigid and stretchable cardiac monitoring devices are available in Appendix of this paper. Appendix also includes the recycling and incineration data for both devices, as well as the incineration data for the PCB. In cases where data are not readily available in the Ecoinvent 3 database, other sources are sought and assumptions are made. Those exceptional cases and additional assumptions are described in the subsections below.

#### Raw material extraction

The life cycle inventory (LCI) data for raw materials for both the products are taken from the Ecoinvent 3 database whenever available. For the materials where the data are unavailable, the LCI data from previously published works are used. For instance, the LCI data for silver nitrate in a rigid device are taken from the study by Bafana et al. (2018). For LCI data of Galinstan in the stretchable device, the data of bulk materials like gallium, indium, and tin from the Ecoinvent 3 database are considered.

#### Manufacturing

The data related to manufacturing processes, such as injection molding and wire drawing in the rigid device, are available in the Ecoinvent 3 database. The LCI data for roll-to-roll printing in strap fabrication are not available in the database or any existing LCA studies. Hence, only the electricity and the raw materials consumed in this step are considered. For the manufacturing processes of the stretchable device, the energy and compressed air consumed, calculated from the equipment rating, are considered due to the unavailability of their LCI data in any database or previous studies.

#### Usage

It has been stated earlier that this study does not include the use phase due to the fact that the use phase in this case primarily involves the energy consumed from the batteries used in both devices, which is identical.

#### End of Life

In the case of the rigid device, it is assumed that the ABS and metallic wires are recycled. The strap, PCB, and battery are incinerated as hazardous industrial waste. Similarly, in the case of the stretchable device, the materials, such as PDMS, polyethylene (PE), and Galinstan, are recycled, while the PCB and battery are incinerated. The emissions to air due to incineration of the PCB in both devices are taken from the report of the US Environmental Protection Agency (2015). For the detailed inventory analysis and LCA modeling, refer to Appendix section in this paper.

### Impact assessment method, assumptions & limitations

Recipe 2016 Midpoint (Hierarchist) is chosen as the impact assessment method due to its strong relation to the environmental flows and a relatively low level of uncertainty (Huijbregts et al. 2016). The environmental burden is expressed in all its eighteen (18) impact categories. The Recipe 2016 Endpoint (Hierarchist) method is used for comparing different life cycle phases of both products. Both devices are assumed to be manufactured and disposed of in Sweden. As mentioned in the previous section, the use and transportation phases have been excluded from this study.

## Results, discussion, and sensitivity analysis

### Results and discussion

**Fig. 5:**
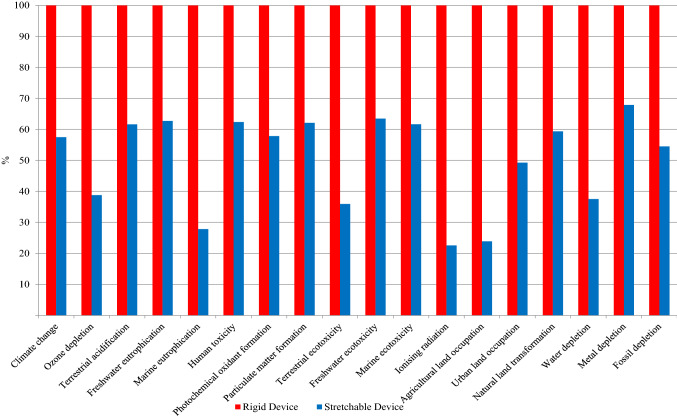
Comparative LCA results of rigid and stretchable devices

Based on the LCI data, a comparative LCA of both the products is carried out using SimaPro. The results of this study are presented in Fig. 5.

**Fig. 6:**
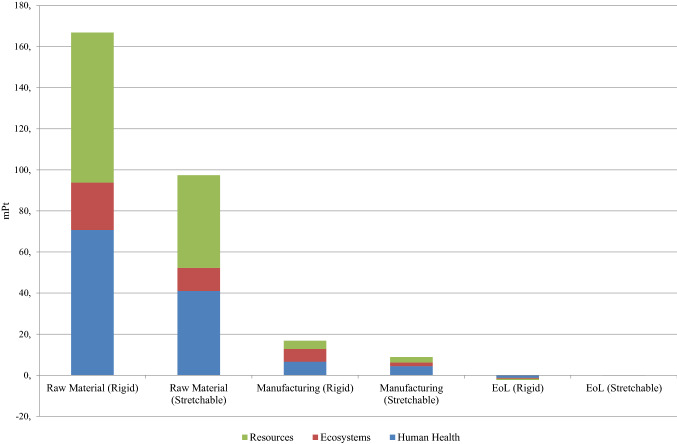
Single score comparison of different life cycle stages of the rigid and stretchable devices

As Fig. 5 shows, the stretchable cardiac monitoring device has lower environmental impacts in all eighteen (18) impact categories compared to the rigid device. From the single score comparison of different life cycle stages as illustrated in Fig. 6, it is clear that the raw material phase in both the devices is the primary contributor to the environmental impacts.

At the raw material extraction stage, it is clear that the stretchable device has less impact than the rigid device. This is attributed to the lower amount of raw materials consumed in the stretchable device, especially for the PWB used in PCB production. The rigid device consumes more PWB than the stretchable device. The manufacturing phase is another significant contributor for both the devices. The higher amount of electricity consumed in the manufacturing processes of the rigid device is the reason behind its higher figure than the stretchable device. The end of life phase of the rigid device has more positive environmental returns than that of the stretchable device due to a higher amount of material being recycled. However, the contribution from the end of life phases is negligible in both cases.

**Fig. 7:**
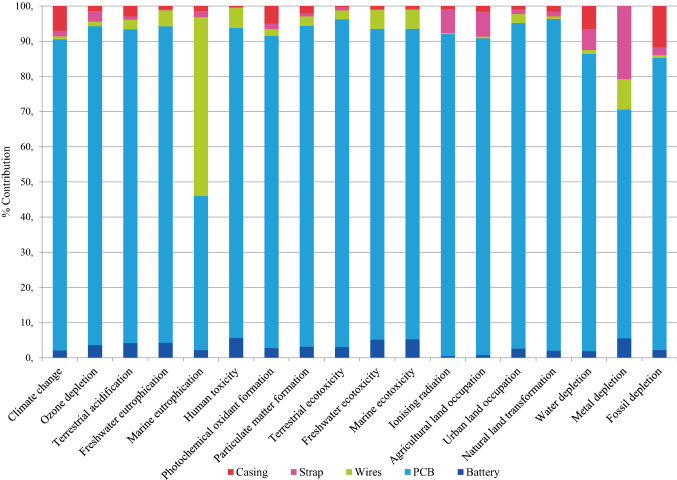
Environmental profile of one rigid device unit

**Fig. 8:**
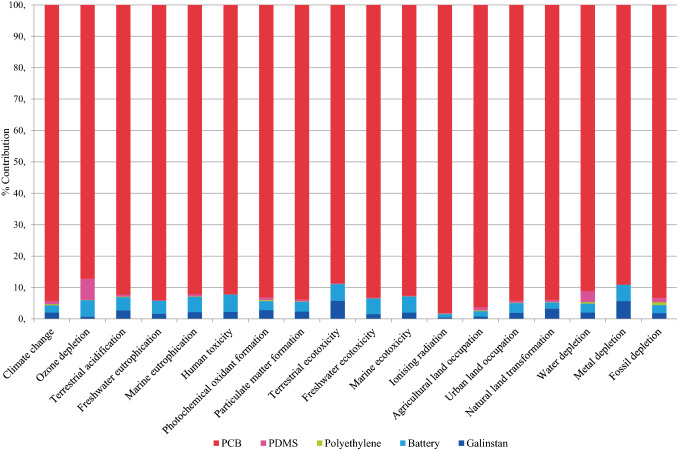
Environmental profile of one stretchable device unit

From the process contribution analysis of the raw materials phase, it is clear that the PCB is the environmental hot spot, i.e., the largest contributor in most of the impact categories. Figures 7 and 8 show the contributions of different components concerning the environmental impacts of rigid and stretchable devices.

In the rigid cardiac monitoring device, the copper consumed in the manufacturing of wires is the largest contributor to the marine eutrophication category (51%). The copper, along with silver used in the strap, is responsible for 9% and 21% of metal depletion, respectively. The ABS casing is another noticeable contributor to fossil depletion (12%), water depletion (7%), and climate change (7%).

In the stretchable cardiac monitoring device, the PCB accounts for more than an 85% share in all the impact categories. PDMS causes 7% of the emissions in ozone depletion. LMA contributes 6% each in terrestrial ecotoxicity and metal depletion. The battery has a 5% share in each of the areas of ozone depletion, human toxicity, terrestrial ecotoxicity, marine ecotoxicity, and metal depletion. The contribution of polyethylene is negligible in all the impact categories (less than 1%).

Looking deeper into the manufacturing phase and considering the time required to manufacture each device, the consumed energy and compressed air used are presented in Tables 2 and 3.

**Table 2:** Energy and compressed air consumed in the manufacturing of the rigid device

Process	Power (W)	Time (min)	Energy (Wh)	Compressed air (liters)
Injection molding	1480	1	24.67	
Roll-to-roll printing	5000	1	83.33	
Wire drawing	7200	1	120	
Loader	300	1	5	
Jet printing	3000	1	50	250
Pick & place	1500	1	25	
Reflow	10,000	5	833.33	
Unloader	300	1	5	
Total			1146.33	250

**Table 3:** Energy and compressed air consumed in the manufacturing of the stretchable device

Process	Power (W)	Time (min)	Energy (Wh)	Compressed air (liters)
Loader	300	1	5	
Dispenser	2000	1	33.33	
Jet printing	3000	1	50	250
Pick & place	1500	1	25	
Dispense	2000	1	33.33	
Unloader	300	1	5	
Total			151.66	250

It is evident from Tables 2 and 3 that the manufacturing of the rigid device consumes more energy (Wh) than the manufacturing of the stretchable device. The reflow process, which is not included in the manufacturing of the stretchable device, is a major contributor to this difference. The manufacturing phase of the rigid device causes nearly twice the environmental impacts than the proposed manufacturing process for the stretchable device as shown in Fig. 6.

From this LCA study, it is clear that the stretchable cardiac monitoring device causes lesser environmental impacts than the rigid cardiac monitor device. The main reason behind this is that the stretchable device consumes approximately six (6) times lesser amount of raw material for PCB and nearly seven (7) times lesser electricity in manufacturing than the rigid device.

### Sensitivity analysis

From the interpretation of LCA results, it is clear that the environmental performance of the cardiac monitoring device is influenced by the raw materials and the energy consumed in their manufacturing process. A sensitivity analysis, included in Appendix section, is carried out to determine how the impact assessment results vary if the raw material, electricity, and compressed air inputs are changed. Each input is varied by + 10% and − 10%, and the changes in impact categories are calculated with reference to the baseline. The sensitivity analysis clearly shows that the variation of the input of PCB material in both devices has a comparatively higher influence on all the impact categories. For instance, in the rigid device, the variation of ± 10% in PCB material amount showed a change of ± 8% in the terrestrial ecotoxicity impact category. Similarly, in the stretchable device, the variation of ± 10% in PCB material input showed a change of ± 7.9% in the climate change impact category.

## Conclusion

A comparative life cycle assessment of stretchable and rigid electronics-based cardiac monitoring devices is conducted to establish which device is better from an environmental point of view. Furthermore, this research compares the environmental impacts of the manufacturing processes of stretchable and rigid electronics when applied to cardiac monitoring device as a representative electronic device.

This study concludes that the stretchable electronics-based device causes significantly lower environmental impacts in comparison with the rigid electronics-based device in all eighteen (18) impact categories defined by the Recipe 2016 Midpoint (Hierarchist) method. The improved environmental performance of the stretchable electronics-based device is primarily linked to the lower usage of raw materials due to innovative methods, such as the utilization of LMA to connect electronic components in and to the PCB reducing the required amount of raw materials.

The study also concludes that the manufacturing process of stretchable electronics has lower environmental impacts. The higher environmental impact of the manufacturing phase of rigid electronics can be attributed to a higher amount of electricity consumed due to manufacturing steps like the reflow phase, injection molding, and roll-to-roll printing.

This study adopted some simplifications due to a lack of field and research data. One of the simplifications implemented is the exclusion of the transportation phase. Since the stretchable device is in the prototype phase, the supply chain of this device is not in place, and therefore, no data are available.

Furthermore, the review presented in this paper indicates that there is very little work on the LCA of stretchable electronics. In this respect, this work is novel but also in its infancy. Further research, as well as more LCA studies, is needed to strengthen the results of this research.

## Data Availability

Not applicable.

## Appendix 1

The results of the sensitivity analysis are shown in Tables

**Table 4:** Sensitivity analysis results for rigid cardiac monitoring device

Input/impact categories	ABS		Battery		PCB		Metal wires		Strap		Electricity		Compressed air	
Variation(%)	+ 10.0	−10.0	+ 10.0	−10.0	+ 10.0	−10.0	+ 10.0	−10.0	+ 10.0	−10.0	+ 10.0	−10.0	+ 10.0	−10.0
Climate change	0.8	−0.7	0.2	−0.2	4.8	−4.7	0.1	0.1	0.1	−0.1	3.0	−3.0	7.0	−7.0
Ozone depletion	0.0	−0.9	0.0	−0.9	5.4	−6.3	0.0	−0.9	0.0	0.0	4.5	−4.5	5.5	−5.5
Terrestrial acidification	0.3	−0.3	0.5	−0.3	4.5	−4.4	0.3	−0.3	0.1	0.0	9.4	−9.4	0.6	−0.6
Freshwater eutrophication	0.0	0.0	0.0	−0.8	4.1	−4.1	0.0	−0.8	0.0	0.0	3.2	−3.2	6.8	−6.8
Marine eutrophication	0.1	−0.1	0.3	−0.3	2.3	−2.3	5.4	−5.3	0.1	−0.1	1.5	−1.5	8.5	−8.5
Human toxicity	0.0	0.0	0.5	−0.5	4.2	−4.2	0.5	−0.5	0.0	0.0	3.2	−3.2	6.8	−6.8
Photochemical oxidant formation	0.6	−0.6	0.3	−0.3	4.5	−4.5	0.3	−0.3	0.3	0.0	1.3	−1.3	8.7	−8.7
Particulate matter formation	0.3	0.0	0.3	−0.3	4.5	−4.5	0.3	−0.3	0.3	0.0	0.6	−0.6	9.4	−9.4
Terrestrial ecotoxicity	0.0	0.0	0.3	−0.3	8.0	−8.0	0.3	−0.3	0.0	0.0	5.5	−5.5	4.5	−4.5
Freshwater ecotoxicity	0.0	−0.3	0.5	−0.8	4.0	−4.0	0.5	−0.8	0.0	−0.3	0.6	−0.6	9.4	−9.4
Marine ecotoxicity	0.3	0.0	0.8	−0.5	4.5	−4.2	0.8	−0.5	0.0	0.0	0.3	−0.3	9.7	−9.7
Ionizing radiation	0.9	0.0	0.0	0.0	5.4	−4.5	0.0	0.0	0.9	0.0	0.3	−0.3	9.7	−9.7
Agricultural land occupation	0.5	−0.7	0.2	−0.4	4.3	−4.5	0.0	−0.4	0.9	0.0	1.3	−1.3	8.7	−8.7
Urban land occupation	0.0	0.0	0.0	−0.8	4.8	−5.6	0.0	−0.8	−0.8	−0.8	0.8	−0.8	9.2	−9.2
Natural land transformation	0.0	−0.6	0.0	−0.6	4.4	−5.1	0.0	0.0	0.0	0.0	9.0	−9.0	1.0	−1.0
Water depletion	1.3	−0.8	0.5	0.0	5.0	−4.4	0.4	0.1	0.4	−0.2	1.8	−1.8	8.2	−8.2
Metal depletion	0.5	0.0	0.9	0.0	1.9	−0.9	−0.9	−0.9	2.8	−1.9	2.2	−2.2	7.8	−7.8
Fossil depletion	1.3	−1.3	0.4	0.0	4.7	−4.3	0.0	0.0	0.4	0.0	8.1	−8.1	1.9	−1.9

^4^ and

**Table 5:** Sensitivity analysis results for stretchable cardiac monitoring device

Input/impact categories	Galinstan		Battery		Polythene		PDMS		PCB		Electricity		Compressed air	
Variation(%)	+ 10	−10	+ 10	−10	+ 10	−10	+ 10	−10	+ 10	−10	+ 10	−10	+ 10	−10
Climate change	0.2	−0.2	0.2	−0.2	0.0	0.0	0.2	0.0	7.9	−7.9	0.2	−0.2	1.5	−1.5
Ozone depletion	0.0	0.0	−4.4	−0.5	0.0	0.0	0.8	−0.6	6.8	−6.7	1.1	−0.9	1.1	−0.9
Terrestrial acidification	0.2	−0.2	0.5	−0.2	0.0	0.0	0.0	0.0	7.7	−7.7	0.2	0.0	1.4	−1.4
Freshwater eutrophication	0.1	−0.1	0.4	−0.4	0.0	0.0	0.0	0.0	7.8	−7.8	0.0	0.0	1.6	−1.6
Marine eutrophication	0.5	0.0	0.5	0.0	0.0	0.0	0.5	0.0	7.6	−7.1	0.5	0.0	1.9	−1.4
Human toxicity	0.8	0.0	0.8	0.0	0.0	0.0	0.8	0.0	7.6	−6.9	0.8	0.0	2.3	−1.5
Photochemical oxidant formation	0.5	0.0	0.5	0.0	0.5	0.0	0.5	0.0	8.4	−7.9	0.5	0.0	1.4	−0.9
Particulate matter formation	0.0	0.0	0.5	−0.5	0.0	0.0	0.0	0.0	7.9	−7.9	0.0	0.0	1.0	−1.0
Terrestrial ecotoxicity	0.0	−0.9	0.0	−0.9	0.0	0.0	0.0	0.0	7.0	−7.8	0.0	0.0	0.9	−1.7
Freshwater ecotoxicity	0.0	−0.4	0.4	−0.7	0.0	0.0	0.0	0.0	7.1	−7.1	0.0	0.0	2.1	−2.5
Marine ecotoxicity	0.4	0.0	0.7	−0.4	0.0	0.0	0.0	0.0	7.2	−6.9	0.0	0.0	2.2	−2.2
Ionizing radiation	0.0	0.0	0.0	0.0	0.0	0.0	0.0	0.0	4.3	−4.3	4.3	−4.3	1.4	−1.4
Agricultural land occupation	0.2	0.0	0.2	−0.2	0.0	0.0	0.2	0.0	4.6	−4.6	3.7	−3.7	1.5	−1.5
Urban land occupation	0.3	−0.1	0.4	−0.3	0.0	0.0	0.1	0.0	7.9	−7.9	0.4	−0.3	1.3	−1.2
Natural land transformation	0.9	0.0	0.9	0.0	0.0	0.0	0.9	0.0	8.5	−7.9	0.9	0.0	0.9	0.0
Water depletion	0.2	−0.2	0.3	−0.3	0.0	0.0	0.2	−0.3	6.3	−6.5	1.0	−1.1	1.6	−1.8
Metal depletion	0.4	−0.6	0.4	−0.6	0.0	0.0	0.0	0.0	8.2	−8.3	0.0	−0.1	0.6	−0.7
Fossil depletion	0.0	−0.7	0.0	−0.7	0.0	−0.7	0.0	−0.7	7.4	−8.1	0.0	−0.7	0.7	−1.5

^5^.

## Appendix 2

The life cycle inventory data for the rigid and stretchable cardiac monitoring device are listed in Tables 6 and Table 9. The recycling and incineration data for both devices, as well as the incineration data for PCB, are included in this appendix in Tables 7, 8, 10, and 11.

**Table 6:** LCI data for the rigid cardiac monitoring device

Component	Inputs	Amount	Unit	Source
ABS Casing	Acrylonitrile butadiene styrene copolymer {RER}| production | Alloc Def. U	12.2616	g	Ecoinvent 3 database
	Injection molding {RER}| processing | Alloc Def. U	12.2616	g	Ecoinvent 3 database
Battery	Battery cell. Li-ion {GLO}| market for | Alloc Def. U	2.8049	g	Ecoinvent 3 database
PCB	Printed wiring board for surface mounting. Pb free surface {GLO}| market for | Alloc Def. U	9.5	cm^2^	Ecoinvent 3 database
	Resistor. surface-mounted {GLO}| production | Alloc Def. U	25.6	mg	Ecoinvent 3 database
	Capacitor. for surface-mounting {GLO}| production | Alloc Def. U	99.9	mg	Ecoinvent 3 database
	Integrated circuit. memory type {GLO}| production | Alloc Def. U	340	mg	Ecoinvent 3 database
	Transistor. surface-mounted {GLO}| production | Alloc Def. U	666	mg	Ecoinvent 3 database
Metallic wires	Steel. unalloyed {RER}| steel production. converter. unalloyed | Alloc Def. U	1.1711	g	Ecoinvent 3 database
	Bronze {RoW}| production | Alloc Def. U	0.1239	g	Ecoinvent 3 database
	Copper {RER}| production. primary | Alloc Def. U	1.7947	g	Ecoinvent 3 database
	Wire drawing. copper {RER}| processing | Alloc Def. U	1.9186	g	Ecoinvent 3 database
	Wire drawing. steel {RER}| processing | Alloc Def. U	1.1711	g	Ecoinvent 3 database
FStrap	Polyvinylchloride. bulk polymerized {RER}| polyvinylchloride production. bulk polymerization | Alloc Def. U	0.1837	g	Ecoinvent 3 database
	Paper. woodfree. coated {RER}| paper production. woodfree. coated. at non-integrated mill | Alloc Def. U	0.3225	g	Ecoinvent 3 database
	Packaging film. low density polyethylene {RER}| production | Alloc Def. U	0.5604	g	Ecoinvent 3 database
	Polyurethane. flexible foam {RER}| production | Alloc Def. U	1.008	g	Ecoinvent 3 database
	Silver nitrate	0.7854	g	(Bafana et al. 2018)
	Acrylic binder. without water. in 34% solution state {RER}| acrylic binder production. product in 34% solution state | Alloc Def. U	1.008	g	Ecoinvent 3 database
Electricity	Electricity. high voltage {SE}| production mix | Alloc Def. U	1001.66	Wh	Ecoinvent 3 database
Compressed air	Compressed air. 1000 kPa gauge {RER}| compressed air production. 1000 kPa gauge. < 30 kW. average generation | Alloc Def. U	250	liters	Ecoinvent 3 database

**Table 7:** Recycling data for the rigid cardiac monitoring device

Materials and/or waste types separated from waste stream	Material/Waste type	Percentage
Mixed plastics (waste treatment) {GLO}| recycling of mixed plastics | Alloc Def. U	Acrylonitrile butadiene styrene copolymer {GLO}| market for | Alloc Def. U	100%
Steel and iron (waste treatment) {GLO}| recycling of steel and iron | Alloc Def. U	Steel. unalloyed {RoW}| steel production. converter. unalloyed | Alloc Def. U	100%
Aluminum (waste treatment) {GLO}| recycling of aluminum | Alloc Def. U	Copper {RER}| production. primary | Alloc Def. U	100%
Aluminum (waste treatment) {GLO}| recycling of aluminum | Alloc Def. U	Bronze {RoW}| production | Alloc Def. U	100%

**Table 8:** Incineration data for the rigid cardiac monitoring device

Materials and/or waste types separated from waste stream	Material/Waste type	Percentage
PCB Incineration	Others	100%
Hazardous waste for incineration {Europe without Switzerland}| treatment of hazardous waste. hazardous waste incineration | Alloc Def. U	All waste types	100%

**Table 9:** LCI Data for the stretchable cardiac monitoring device

Component	Input	Amount	Unit	Source
Galinstan	Gallium. in Bayer liquor from aluminum production {GLO}| production | Alloc Def. U	0.1511	g	Ecoinvent 3 database
	Tin {RER}| production | Alloc Def. U	0.02206	g	Ecoinvent 3 database
	Indium {RER}| production | Alloc Def. U	0.04743	g	Ecoinvent 3 database
Battery	Battery cell. Li-ion {GLO}| market for | Alloc Def. U	1.6284	g	Ecoinvent 3 database
Packaging film	Packaging film. low density polyethylene {RER}| production | Alloc Def. U	0.6374	g	Ecoinvent 3 database
PDMS	Silicone product {RER}| production | Alloc Def. U	1.6205	g	Ecoinvent 3 database
PCB	Printed wiring board. for surface mounting. Pb free surface {GLO}| market for | Alloc Def. U	1.69	cm^2^	Ecoinvent 3 database
	Resistor. surface-mounted {GLO}| production | Alloc Def. U	25.6	mg	Ecoinvent 3 database
	Capacitor. for surface-mounting {GLO}| production | Alloc Def. U	99.9	mg	Ecoinvent 3 database
	Integrated circuit. memory type {GLO}| production | Alloc Def. U	340	mg	Ecoinvent 3 database
	Transistor. surface-mounted {GLO}| production | Alloc Def. U	666	mg	Ecoinvent 3 database
Electricity	Electricity. high voltage {SE}| production mix | Alloc Def. U	151.66	Wh	Ecoinvent 3 database
Compressed Air	Compressed air. 1000 kPa gauge {RER}| compressed air production. 1000 kPa gauge. < 30 kW. average generation | Alloc Def. U	250	liters	Ecoinvent 3 database

**Table 10:** Incineration data for the stretchable cardiac monitoring device

Materials and/or waste types separated from waste stream	Material/Waste type	Percentage
PCB Incineration	Others	100%
Hazardous waste for incineration {Europe without Switzerland}| treatment of hazardous waste. hazardous waste incineration | Alloc Def. U	All waste types	100%

**Table 11:** Incineration data for the PCB (per kg) (United States Environmental Protection Agency 2015)

Emissions to air	Amount	Unit
Carbon monoxide	0.12	kg
Particulates. unspecified	23	g
Carbon dioxide	1.08	kg
Polycyclic organic matter. unspecified	11	g

## Footnotes

1 https://www.sintec-project.eu/.

2 https://www.indium.com/
